# Spatial and temporal trends of cesarean deliveries in Uganda: 2012–2016

**DOI:** 10.1186/s12884-019-2279-6

**Published:** 2019-04-16

**Authors:** Emily B. Atuheire, Denis Nixon Opio, Daniel Kadobera, Alex R. Ario, Joseph K. B. Matovu, Julie Harris, Lilian Bulage, Blandina Nakiganda, Nazarius Mbona Tumwesigye, Bao-Ping Zhu, Frank Kaharuza

**Affiliations:** 1 Uganda Public Health Fellowship Program, P.O. Box 7272, Kampala, Uganda; 2 Reproductive Health Division, Ministry of Health, Kampala, Uganda; 3 Makerere University School of Public Health, Kampala, Uganda; 4 US Centers for Disease Control and Prevention, Atlanta, USA; 5 African Field Epidemiology Network, Kampala, Uganda; 6 US Centers for Disease Control and Prevention, Kampala, Uganda

**Keywords:** Trends, Cesarean deliveries, Cesarean section rate, Uganda

## Abstract

**Background:**

Cesarean section (CS) is an important intervention in complicated births when the safety of the mother or baby is compromised. Despite worldwide concerns about the overutilization of CS in recent years, many African women and their newborns still die because of limited or no access to CS services. We evaluated temporal and spatial trends in CS births in Uganda and modeled future trends to inform programming.

**Methods:**

We performed secondary analysis of total births data from the Uganda National Health Management Information System (HMIS) reports during 2012–2016. We reviewed data from 3461 health facilities providing basic, essential obstetric and emergency obstetric care services in all 112 districts. We defined facility-based CS rate as the proportion of cesarean deliveries among total live births in facilities, and estimated the population-based CS rate using the total number of cesarean deliveries as a proportion of annual expected births (including facility-based and non-facility-based) for each district.

We predicted CS rates for 2021 using Generalised Linear Models with Poisson family, Log link and Unbiased Sandwich Standard errors. We used cesarean deliveries as the dependent variable and calendar year as the independent variable.

**Results:**

Cesarean delivery rates increased both at facility and population levels in Uganda. Overall, the CS rate for live births at facilities was 9.9%, increasing from 8.5% in 2012 to 11% in 2016. The overall population-based CS rate was 4.7%, and increased from 3.2 to 5.9% over the same period. Health Centre IV level facilities had the largest annual rate of increase in CS rate between 2012 and 2016. Among all 112 districts, 80 (72%) had a population CS rate below 5%, while 38 (34%) had a CS rate below 1% over the study period. Overall, Uganda’s facility-based CS rate is projected to increase by 36% (PRR 1.36, 95% CI 1.35–1.36) in 2021 while the population-based CS rate is estimated to have doubled (PRR 2.12, 95% CI 2.11–2.12) from the baseline in 2016.

**Conclusion:**

Cesarean deliveries are increasing in Uganda. Health center IVs saw the largest increases in CS, and while there was regional heterogeneity in changes in CS rates, utilization of CS services is inadequate in most districts. We recommend expansion of CS services to improve availability.

## Background

Cesarean section (CS) is an important intervention in complicated births when the safety of the mother or baby is compromised [1]. Globally, approximately 15 in 100 pregnant women require CS to prevent poor outcomes for them and/or their newborns [2, 3]. A facility or population-level CS rate below 5% suggests that women lack access to emergency obstetric care services, while a 10–15% rate is generally accepted as optimal [4, 5]. Worldwide, CS rates have increased tremendously in recent years, especially among high-income countries, raising concerns about over-utilization of CS without added benefits [6]. However, in Sub-Saharan Africa, where two-thirds of the world’s 302,000 maternal deaths occur annually, the CS rate is the lowest in the world (7.3%) [7] and women and their newborns often end up dying or sustaining unnecessary injuries due to limited access to and underutilization of CS services [8]. Several factors, including weak health systems, a shortage of human resources, inadequate financial resources, long distances to health facilities, poor transport systems, poverty, and low literacy levels have been documented as barriers to obtaining CS [9].

Uganda is struggling with a high maternal mortality ratio (MMR), estimated at 336 per 100,000 live births in 2016; this translates into a lifetime risk of maternal death of 1 in 47 [10]. To address this, the government has made deliberate efforts to increase availability, quality, access to, and utilization of emergency obstetric care services to manage and treat complications of pregnancy, labour, and delivery [11, 12]. The Uganda Ministry of Health (UMOH) uses the CS rate as an indicator for measuring these characteristics, and for measuring functionality of the health service system [4, 13]. A study in 2011 estimated that 5.2% of all Ugandan women delivered their babies via CS, up from 3.1–3.6% in 2006 [6, 7, 14].

Uganda’s healthcare system comprises multiple levels of care, including health centers II, III, and IV, general hospitals, and referral hospitals. For care related to childbirth, health center IIs and small clinics are mandated to provide essential obstetric care including antenatal care, preventive services, and treatment for common illnesses. Health center IIIs provide a wider range of services, including normal deliveries and first aid for complications of pregnancy, labour, and delivery. Health center IIIs also provide a set of six lifesaving interventions (signal functions) including parenteral antibiotics, oxytocic drugs, anticonvulsants, assisted vaginal delivery, manual removal of placenta, and removal of retained products for women with pregnancy-related complications (also called basic emergency obstetric care (BEmOC)). Health center IVs act as mini-hospitals and are the first referral level for low- or moderate-risk pregnant women. Both health center IVs and hospitals are mandated to provide comprehensive emergency obstetric care (CEmOC), including cesarean section and blood transfusion services as well as the lifesaving interventions provided at lower health centers. Health center IVs and hospitals also refer women with high-risk pregnancies to regional Referral Hospitals [15].

Despite these mandates, comprehensive obstetric care is not available in many health facilities that are mandated to offer it in Uganda [13, 15]. Although there is evidence for improvements in skilled birth attendance, from 57% in 2011 to 74% in 2016, and health center IVs are increasingly providing CS services [16], there are few recent data on CS rates in Uganda [17–19]. We ascertained temporal and spatial trends in CS in Uganda, and modeled predictions for Cesarean section births for 2021 in the Ugandan population and in health facilities to inform programming.

## Methods

### Study design

We performed secondary data analysis of total births obtained from the Uganda National Health Management Information System (HMIS) reports between 2012 and 2016. The HMIS is a nationwide surveillance system that monitors patterns of morbidity and mortality and health care services, including maternal and child health. Our sources of data were the monthly HMIS reports for outpatient and inpatient maternity attendances. The average reporting rate over the study period was 87% (range 74–94%), covering both public and private facilities.

### Data extraction and calculation of cesarean section rates

We reviewed total births data including normal and cesarean deliveries reported from 3461 health facilities (public and private) designated to provide maternity care. Of the 3461 facilities, 1976 were health center IIs providing essential obstetric care; 1091 were health center IIIs providing basic emergency obstetric care (BEmOC), and 394 facilities provided comprehensive emergency obstetric care (CEmOC), including 181 health center IVs, 123 general hospitals, 14 referral hospitals, and 76 small clinics (Table 1).

**Table 1:** Distribution of CS in all facilities providing total births data: Uganda, 2012 and 2016

Health facilities in each category (n) and proportion providing CS (%)		
Facility level	2012	2016
Comprehensive emergency obstetric care		
Referral Hospital	14 (100%)	15 (100%)
General Hospital	123 (80%)	140 (76%)
Health center IV	181 (32%)	181 (72%)
Private Small Clinics	76 (26%)	227 (4.8%)
Basic emergency obstetric care^a^		
Health Center III	1091 (0.91%)	1144 (2.6%)
Essential obstetric care^b^		
Health Center II	1976 (0%)	2610 (0%)
All facilities	3461 (5.8%)	4317 (6.8%)

*CS* Cesarean section

^a^Some Health center IIIs are providing CS in addition to their designated role as basic emergency obstetric care centers

^b^Some Health center IIs are conducting normal deliveries, though they are only intended to offer antenatal care services

N refers to the number of facilities in that category. Percentage refers to the proportion of all facilities in that category providing CS

We defined facility-based CS rate as the proportion of CS deliveries among total live births in the facility. To estimate the facility-based CS rate, we used Cesarean births data from CEmOC sites [5, 20]. Data from lower health centers were not included for this calculation as these facilities do not provide CS services [21].

In estimating the population-based CS rate, we used the aggregated Cesarean deliveries per district as the numerator, and multiplied the crude birth rate (4.85%) by the annual population estimates per district (2014 census) to obtain the expected births (denominator) [5, 22, 23].

### Temporal and spatial trend analysis

We described trends for 2012–2016 and calculated the absolute increase in CS rate by subtracting the earliest CS rate (2012) from the latest (2016) [24, 25]. We compared facility-based CS rates across CEmOC facilities and health service regions. For population-based CS rates, we calculated overall trends at the national level and by health service region (i.e., referral zones comprising clusters of districts that form the catchment area/population served per referral hospital) [26, 27].

We used Generalised Linear Models with Poisson family, Log link, and Unbiased Sandwich Standard errors to test the significance of CS trends. We used CS as the dependent variable and calendar year as the independent variable to generate prevalence rate ratios (PRRs) for the period 2012–2016 [28, 29]. We interpreted the resulting PRR estimates as the annual rate of increase in the CS rate and predicted the CS rates for 2021 based on the median rate for 2012–2016. We used Geographic Information Software (QGIS 2.14.8) to display the spatial prevalence of CS in Uganda, and STATA 14 to perform the analysis.

Because our study used routine program surveillance data reported by health facilities for program monitoring and evaluation and the analyzed data were also aggregated with no individual patient identifiers, we did not seek for ethical approval. However, we sought and obtained permission to use the data from the Uganda Ministry of Health. The US Centers for Disease Control and Prevention (CDC) also provided the non-research determination (NRD 2017–201) for non-human subjects. Analyzed data were also aggregated with no individual patient identifiers. Data were only accessed by the study team.

## Results

### Trends in facility-based cesarean section rates in Uganda, Jan 2012-Dec 2016

There were 4,256,784 total births, of which 4,038,137 were live births, from 3461 health facilities across the 112 districts in Uganda between 2012 and 2016. The proportion of all facilities providing CS services increased from 5.8% in 2012 to 6.8% in 2016 while the proportion of health center IVs providing CS more than doubled (Table 1).

**Fig. 1:**
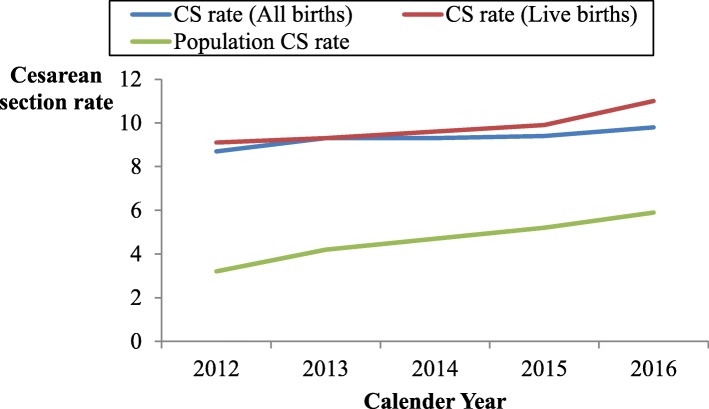
Line graph showing temporal changes in CS rates in Uganda between 2012 and 2016. The graph shows the five year trend for CS rates at facility level for both total and live births, and population CS rates from 2012 to 2016

Of all live births, 398,113 were cesarean deliveries, for an overall facility-based CS rate of 9.9% for live births. The annual facility-based CS rate among live births increased from 9.1% in 2012 to 11% in 2016 (Fig. 1).

The CS rate for CEmOC facilities increased from 19% in 2012 to 22% in 2016. However, the CS rates across the different levels of care varied markedly. During 2012–2016, the highest CS rates occurred in general hospitals (22–32%), followed by referral hospitals (20–25%), and health center IV facilities (4.0–8.0%). Health centers IV level had the highest annual increase in CS rates, followed by general hospitals (Table 2).

**Table 2:** Changes in facility-based CS rates, by facility type and referral regions, Uganda, 2012–2016

CS rates by level of care	CS rate 2012–2016	Median CS rate	PRR^a^2012–2016^a^	PRR for CS2021^b^
Health center IV	4.0–8.0	5.8	1.18 (1.17–1.19)	2.30 (2.28–2.32)
Gen. Hospital	22–32	25	1.10 (1.09–1.10)	1.58 (1.57–1.58)
Referral Hospitals	20–25	23	1.08 (1.07–1.08)	1.45 (1.44–1.45)
All CEmOCs	19–22	19	1.09 (1.09–1.09)	1.52 (1.51–1.52)
Health service referral regions				
Fort Portal	11–26	13	1.25 (1.24–1.25)	3.00 (2.97–3.03)
Mulago	10–14	13	1.09 (1.09–1.10)	1.55 (1.54–1.55)
Lira	4.8–5.5	5.5	1.06 (1.05–1.08)	1.36 (1.35–1.37)
Gulu	4.5–6.0	5.5	1.06 (1.05–1.07)	1.34 (1.33–1.34)
Hoima	8.1–10	10	1.06 (1.05–1.07)	1.32 (1.31–1.32)
Kabale	11–13	12	1.06 (1.05–1.07)	1.33 (1.32–1.33)
Mbarara	11–15	14	1.05 (1.04–1.05)	1.25 (1.24–1.25)
Jinja	5.2–6.8	6.8	1.04 (1.03–1.04)	1.19 (1.18–1.19)
Arua	7.3–8.3	7.9	1.03 (1.02–1.04)	1.17 (1.16–1.17)
Mbale	5.7–6.8	6.1	1.03 (1.02–1.04)	1.16 (1.15–1.16)
Mubende	10–11	11	1.03 (1.02–1.04)	1.15 (1.14–1.15)
Soroti	4.7–4.5	4.6	0.97 (0.96–0.99)	0.87 (0.86–0.87)
Moroto	5.3–3.9	3.9	0.93 (0.92–0.95)	1.48 (1.47–1.49)
Masaka	17–11	14	0.93 (0.92–0.93)	0.68 (0.66–0.70)
All Uganda	9.1–11	9.6	1.06 (1.06–1.06)	1.36 (1.35–1.36)

*CS* Cesarean section, *CEmOC* Comprehensive emergency obstetric care, *PRR* Prevalence Rate Ratio

^a^Estimated PRR is equivalent to the annual rate of increase in the CS rate

^b^Ratio of CS rate in 2021 to CS rate for the baseline period (2012 to 2016)

Facility-based CS rates varied widely across referral regions. Fort Portal (11–26%) region had the largest increase in facility CS rates, while Masaka (17–11%) region had the largest reduction in CS rates (Table 2).

### Trends in population-based cesarean section rates in Uganda, Jan 2012-Dec 2016

The overall population-based CS rate was 4.7%, and increased from 3.2 to 5.9% over the 2012–2016 period (Fig. 1).

All referral regions had significant increases in population-based CS rates over the 5-year period. In 2012, only Masaka (5.2%) and Fort Portal (5.0%) referral regions had CS rates ≥5%. However, in 2016, four additional referral regions had exceeded 5% CS rates. During the 2012–2016 period, Fort Portal region had the largest absolute increase in population-based CS rates, of 9% (PRR = 1.17, 95% CI 1.17–1.18) while Masaka region had the smallest increase, of 1.2% (PRR = 1.07, 95% CI 1.07–1.08). Soroti region had the lowest median rate overall (Table 3).

**Table 3:** Population based CS rates by referral region, Uganda, 2012–2016

Health service referral region	% change in population-based CS rates ^a^	Median CS rate	Estimated PRR for 2012–2016	Projected PRR for CS 2021^b^
Fort Portal	5.0–14	5.9	1.32 (1.31–1.33)	3.88 (3.84–3.93)
Mbale	1.6–3.7	2.9	1.21 (1.20–1.21)	2.34 (2.32–2.36)
Kabale	3.9–8.3	6.6	1.18 (1.17–1.19)	2.23 (2.21–2.25)
Lira	1.5–2.8	2.3	1.17 (1.16–1.19)	2.60 (2.58–2.62)
Jinja	1.7–3.6	3	1.16 (1.15–1.17)	2.34 (2.32–2.36)
Mubende	10–11	12	1.15 (1.14–1.17)	2.04 (2.02–2.06)
Mbarara	3.7–7.4	6	1.14 (1.14–1.15)	1.95 (1.94–1.96)
Hoima	2.9–5.0	4.5	1.14 (1.13–1.15)	1.92 (1.91–1.93)
Arua	2.8–4.6	4	1.14 (1.13–1.15)	1.92 (1.91–1.93)
Mulago	10–14	7	1.14 (1.13–1.14)	1.90 (1.89–1.91)
Gulu	2.1–3.7	3.3	1.12 (1.10–1.13)	1.74 (1.73–1.75)
Moroto	1.3–2.0	1.5	1.12 (1.10–1.15)	1.79 (1.55–2.07)
Masaka	5.2–6.4	6.4	1.07 (1.07–1.08)	1.42 (1.41–1.42)
Soroti	0.99–2.4	1.4	1.00 (0.99–1.01)	1.00 (1.00–1.00)
All Uganda	3.2–5.9	4.6	1.16 (1.15–1.16)	2.12 (2.11–2.12)

*CS* Cesarean section, *PRR* Prevalence Rate Ratio

^a^CS rates for baseline year 2012 and latest year 2016

^b^Ratio of CS rate in 2021 to CS rate for the baseline period (2012 to 2016)

**Fig. 2:**
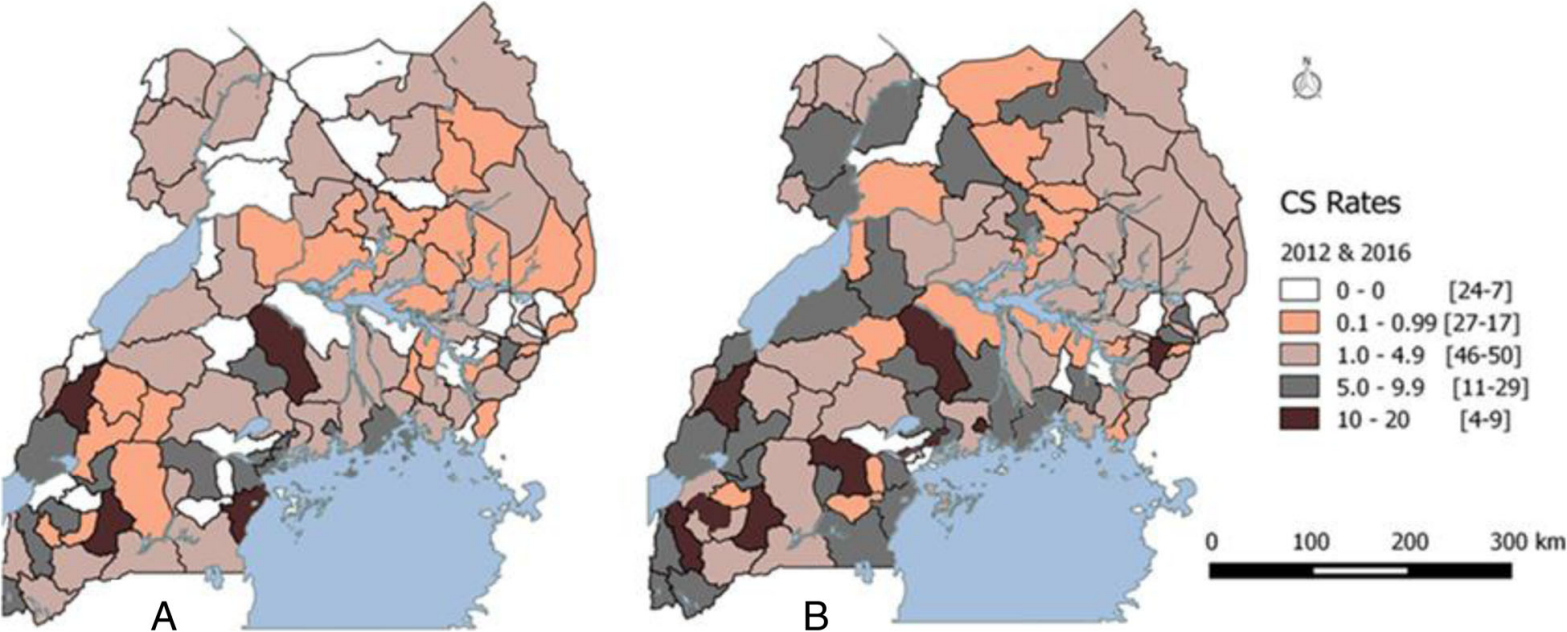
Map of Uganda showing population based CS rates per District in 2012 (Map **a**) & 2016 (Map **b**). The color codes on each map represent the districts categorized by CS rates (range 0, 0.1-.99, 1-4.9, 5-9.9, and 10-20). We generated maps **a** and **b** of Uganda in Fig. 2 using Geographic Information Software (QGIS 2.14.8) to depict the spatial distribution of CS rates at district level during 2012 and 2016

The average population CS rate was below 5% in 80 (72%) districts and below 1% in 38 (34%) districts. In four (3.4%) districts, there were no Cesarean sections offered during the study period. Of the 32 (29%) districts with a CS rate ≥ 5%, eight had an average CS rate above 10% during 2012 to 2016 (Fig. 2).

### Facility-based CS rate projections for 2021

Overall, Uganda’s facility-based CS rate is projected to increase by 36% (PRR 1.36, 95% CI 1.35–1.36) by 2021. Fort Portal referral region is estimated to have a fourfold increase and the highest facility-based CS rates in 2021, while Masaka referral region is projected to have a decrease (PRR 0.68, 95% CI, 0.66–0.70) in CS rates. Health center IVs are projected to double their CS rates (PRR 2.30, 95% CI 2.28–2.32), having the largest increases among CEmOC facilities (PRR 1.52, 95% CI 1.51–1.52) and the smallest increases (PRR 1.45, 95% CI 1.44–1.45) at referral hospitals (Table 2).

### Population CS rate projections for the year 2021

In 2021, Uganda’s population-based CS rate is estimated to have doubled (PRR 2.12, 95% CI 2.11–2.12) from the baseline in 2016. There will be a varied increase in population CS rates across the referral regions in Uganda. Fort Portal referral region is projected to have a threefold increase (the largest across regions) in population-based CS rates (PRR 3.88, 95% CI 3.84–3.93), while Soroti region will have no change from the baseline CS rates (PRR 1.00, 95% CI 1.00–1.00).(Table 3).

## Discussion

We found an increasing trend in cesarean deliveries in Uganda. While some districts have reached the 10–15% population-based CS rate target representing optimal availability and use of CS, many districts remain below this level, indicating a gap in access which still needs to be addressed.

We found variations in population-based CS rates and in the changes in CS rates across the referral regions. Throughout the study period, there were several interventions at national and local levels aimed at increasing CS services, including recruitment of obstetricians, anesthetists, and midwives, as well as refurbishment of facilities to ensure provision of emergency surgical care in target populations [12, 30]. Fort Portal referral region in western Uganda saw the largest absolute increase in population-based CS rates, compared to other referral regions. This may be due to interventions such as the Saving Mothers Giving Life (SMGL) initiative, which aimed at improving safe deliveries and access to quality obstetric services that was implemented in the region during the study period [16]. Soroti referral region had the lowest rates of cesarean deliveries, reflecting a lack of availability of CEmOC in this region during the study period. Previous studies have shown major gaps in the human resources and infrastructure for emergency obstetric care in Soroti region, as well as low utilization of maternal health care services [15, 31]. Expanding SMGL and other similar initiatives to areas still struggling with low CS access, such as Soroti referral region, may be warranted.

Health center IVs had the largest increase in CS rates, followed by general hospitals. This can be attributed to the increasing number of Health center IVs that provided CS services over the study period. The increase at general hospitals might reflect a well-functioning referral system in which lower-level health centers are identifying and referring complicated pregnancies to a higher level for better management [32].

Most districts performed below the 5% minimum CS rate recommended by the World Health Organization for lifesaving benefit, and one-third had a CS rate below 1%. CS services were lacking mostly in districts where the maximum level of care was provided by health center IV facilities. Districts with few or no CS also had a low coverage of CEmOC facilities, representing an important health gap that, if filled, could have an immediate impact [26, 33]. CS rates were higher in districts hosting regional referral hospitals and those with teaching hospitals. These high rates could have been influenced by the wider catchment population for referral hospitals whose services cover several districts or the availability of human resources boosted by the teaching hospitals [21, 34].

The varied increase in CS rates projected for 2021 suggest that the government may need to implement differential levels of interventions across districts, to ensure equitable access to the service by those who need it. In a country where maternal morbidity and mortality remain high, improving availability of CS services is key to reducing mortality and achieving the 2030 sustainable development goals [10]. However, understanding the drivers of – and barriers to - CS is also key to improving programming for maternal and newborn health services [35].

While previous studies have reported CS trends at a national level in Uganda, ours is the first to evaluate CS rates at sub-national/district and facility levels (9, 24). However, we faced the following limitations. First, we used aggregated birth data, thus were unable to adjust for factors such as indications for CS (e.g. Robson criteria) and multiple pregnancies [36]. In addition, we used the 2014 census population estimates to calculate CS rates for the previous years (2012 and 2013) due to inconsistencies in the previous population projections [23]. This may have resulted in underestimation of the annual CS rates arising from exaggerated expected births for 2012 and 2013 as compared to 2014.

## Conclusion

In conclusion, CS rates across sub- regions and districts in Uganda are still low. We recommend expansion of CS services to districts without the service to improve availability and to functionalize more health center IVs to provide CS.

## Abbreviations

BEmOC: Basic emergency obstetric care
CEmOC: Comprehensive emergency obstetric care
CI: Confidence intervals
CS: Cesarean section
HMIS: Health Management Information System
MMR: Maternal mortality ratio
NRD: Non research determination
PRR: Prevalence rate ratio
QGIS: Geographical information software
SMGL: Saving Mothers Giving Life
UMoH: Uganda Ministry of Health
US CDC: United States Centers for Diseases Control and Prevention
